#  Evaluation of E-cadherin Expression in Gastric Cancer and Its Correlation with Clinicopathologic Parameters

**Published:** 2017-04-01

**Authors:** Zhila Torabizadeh, Anahita Nosrati, Seyedeh Neda Sajadi Saravi, Jamshid Yazdani Charati, Ghasem Janbabai

**Affiliations:** 1 MD, Professor, Department of Pathology, School of Medicine, Gut and Liver Research Center, Mazandaran University of Medical Sciences, Sari, Iran; 2 MD, Assistant Professor, Department of Pathology, School of Medicine, Mazandaran University of Medical Sciences, Sari, Iran; 3 MD, Resident, Department of Pathology, School of Medicine, Mazandaran University of Medical Sciences, Sari, Iran; 4 MD, Associate Professor, Department of Biostatistics, School of Public Health, Mazandaran University of Medical Sciences, Sari, Iran; 5 MD, Associate Professor, Department of Internal Medicine, School of Medicine, Gastrointestinal Cancer Research Center, Mazandaran University of Medical Sciences, Sari, Iran

**Keywords:** Gastric cancer, E-cadherin, Clinicopathologic, Immunohistochemistry, Stage

## Abstract

**Background:** Gastric cancer is one of the most common cancers in the world. There are many genomic and molecular factors that cause gastric cancer to occur. Also, many markers that associate with tumor invasiveness have been known.

E-cadherin is a calcium- mediated cell adhesion molecule. In some studies, abnormal expression of E-cadherin has been seen in gastric carcinoma. However, in the studies done there has been some conflicting information about abnormal expression of this marker in a variety of gastric carcinoma and also about the expression of this marker and its correlation with various clinicopathologic factors of tumor.

**Subjects and** **Methods: **A case control study was performed on total or partial gastrectomy tissue samples obtained from 70 patients with gastric cancer and adjacent non-neoplastic tissues. The immunohistochemistry was used to assess E-cadherin expression. The correlation between abnormal E-cadherin expression and tumor histopathology was evaluated in all patients.

**Results: **Among 70 patients who were analyzed, 48.6% showed abnormal E-cadherin expression**.** A significant correlation was seen between abnormal E-cadherin expression and tumor stage, grade, lymph node metastasis, tumor phenotype, tumor type, depth of invasion and age.

**Conclusion: **Abnormal E-cadherin expression is a common phenomenon in gastric cancer. Because there was a strong correlation between abnormal E-cadherin expression and tumor stage, tumor grade, depth of invasion and regional lymph node involvement, this marker may be used as a predictive factor for tumor invasiveness in gastric cancer.

## Introduction

 Gastric cancer is the fourth most common cancer and the second leading cause of death in both sexes in the world.^1^^,^^2^ Gastric cancer is the most common gastrointestinal cancer in Iranian men and the third most common cancer in Iranian women (after breast and colon).^3^^,^^4^ According to the Ministry of Health, gastric cancer is the most common fatal gastrointestinal tract malignancy and the most common cause of death in Iran.^5^^, ^^6^^, ^^7^Since the incidence of this cancer in Iran is twice its incidence in the world, it requires special attention.^4^^,^^5^ In general, initial gastric cancers are often adenocarcinomas, which based on pathologic classification are divided into two types of intestinal and diffuse.^8^

Cadherin is a group of cell adhesion molecules with glycoprotein nature that are located in the membrane of the cell. These molecules are located in combination with other cell adhesion molecules (such as Catenins) and regulate various biological important processes such as cell migration, differentiation, proliferation and cell death (apoptosis).Two types of Cadherins that have been most studied are E-cadherin (epithelial type) and N-Cadherin (neural type).^9^ Epithelial type is a calcium-mediated cell adhesion molecule and its abnormal (low) expression has associated with advanced stages and more aggressive behavior of some cancers including large bowel, lung and prostate.^10^ Also, in various studies, there has been a relationship between the level of E-cadherin expression and the sensitivity of tumor cells to chemotherapy.^11^^,^^12^ This marker is detectable by IHC staining and exists in the normal gastric tissue. In some studies, abnormal expression of E-cadherin is found in gastric carcinoma.^13^ However, in current studies, there are some conflicting information about abnormal expression of this marker in a variety of gastric carcinoma and also about its correlation with various clinicopathologic factors of tumor.

Due to direct correlation between the risk of metastatic disease that is directly related to patient prognosis, and clinicopathologic factors^14^increasing the prevalence of gastric cancer in different communities, including our country, Iran, lack of appropriate treatment response , shortcomings and contradictions that exist in many previous studies as well as insufficient studies in this field, we decided to investigate the expression of E-cadherin in all different kinds of gastric cancer and its correlation with each of mentioned parameters. It is hoped that this study can be a step towards better recognition of gastric cancer behavior and it can also be effective in the way of intervention and follow-up of these patients.

## MATERIALS AND METHODS

 We reviewed the tissue obtained from gastrectomy samples in 70 patients. We obtained two different tissue samples from each patient. One, from the area with malignant cells and one from the area with normal cells. This study was an analytic case-control design and was done on two groups. Group one included tissue samples with cancerous cells and group two included tissue samples with normal cells. It aimed to investigate the correlation between abnormal expressions of E-cadherin and clinicopathologic features of tumor in gastric cancer, on paraffin blocks available at Archives of Pathology in Imam Khomeini Hospital in Sari, Iran. There are 70 samples in both groups.

Sample size was calculated through the formula Pokak. The clinicopathologic parameters that were evaluated in this study included age, gender, tumor type, tumor location, tumor size, tumor phenotype, histological grade, lymph node metastasis (N), distant metastasis (M) and stage.

In order to compare the expression of E-cadherin and intensity of staining in clinicopathologic parameters, the patients were classified according to age, tumor size and lymph node metastasis. The patients' age were divided into two groups (under and above 55 years). Tumor size and lymph node metastasis were divided into three groups (less than 5 cm, 5 to 10 cm and above 10 cm) and four groups (Lack of involvement, 1 to 6 involvement, 7 to 15-involvement, and 15 and more than 15 involvement), respectively.

Patients who had preoperative chemotherapy or radiotherapy were excluded. The patient data were entered in the questionnaire. In order to follow-up patients, their addresses and telephone numbers were used. (All procedures performed in this study were in accordance with the ethical standards of the institution. ethical code: 1393/6/16 )

Tumor blocks or slides that were not suitable for immuno-histochemical staining were omitted from the study.

To continue the study, the needed paraffin blocks were removed from the archive and several slides with hematoxylin – eosin staining were prepared from the areas of tumor and adjacent normal looking tissue. 

During the investigation of slides, in addition to detecting tumor, other microscopic parameters such as depth of invasion, neurovascular invasion and differentiation (histological grade) were also evaluated.

Immunohistochemical staining was performed on the samples for assessing the expression of E-cadherin. The staining step followed the routine process. In order to examine the specificity of immune staining, both positive and negative controls were run at the same time in each experiment.^15^^,^^16^ The slides were studied and reported by two expert pathologists who had no clinicopathologic knowledge of the patients' data, E-cadherin expression and intensity of staining.

To improve the accuracy of diagnosis and to determine the staining of cells, multiple microscopic fields in low and high-power (X100-X400) were examined and the findings were reported according to the estimated percentage of the stained tumor cells.

Staining intensity of E-cadherin was reported on a scale with three grades:

Positive staining in less than 10% of tumor cells.:0

Positive staining between 10% to 90% of tumor cells. :+1

Positive staining Over 90% of the tumor cells.:+2

0 to 1+ grade as low staining and +2 grade as high staining were considered for E-cadherin.^17^

E-cadherin protein was a membranous marker. The obtained results were analyzed by statistical software SPSS (IBM SPSS Statistics 20.0.1). Chi –square, Gama and Fisher's exact tests were used to analyze the correlation between the expression of E-cadherin in gastric cancer and clinicopathologic features. A p-value less than 0.05 was considered as a statistically significant level.

## Results

 A total of 70 patients (49 men and 21 women) were enrolled and evaluated in this study. The clinicopathologic findings in patients are summarized in Table 1. The mean of patients’ age was 65.87 ± 11.28 years. After immunohistochemical staining, E-cadherin expression and intensity of staining in the two groups (case and control) were compared (Table 2).

**Table 1:** Clinicopathologic findings in patients with gastric cancer

**stage**	**Lymph ** **node**	**Neurovas** **cular ** **involvem** **ent**	**Tumor ** **grade**	**Tumor ** **shape**	**Depth of ** **invasion**	**Tumor ** **size**	**Tumor location**	**Tumor ** **Phenotyp** **e**	**Tumor ** **type**	**Gender**	**Age**
20% (n=14) Ia-Ib	40% (n=28) N0	48.6 %(n=34) yes	31.4% (n=22) Well	95.7% (n=67) Ulcerative	2.9% (n=2) T1	62.9% (n=44) <=5 cm	17.1% (n=12) cardia	81.4% (n=57) Intestinal	94.3% (n=66) Adenocar cinoma	70 %(n=49) male	12.9% (n=9) Under55 years
27.1% (n=19) II	27.1% (n=19)N1	51.4% (n=36) no	30% (n=21) moderate	4.3%(n=3) Infiltrative	27.1% (n=19) T2	35.7% (n=25) 5-10cm	4.3% (n=3)fund us	18.6% (n=13) Diffuse	5.7% (n=4) Signet ring carcinoma a	30 % (n=21) Female	87.1% (n=61) above55 years
52.9% (n=37) IIIa-IIIb	32.9% (n=23)N2		38.6% (n=27)po or		70% (n=49) T3	1.4% (n=1) >10cm	10% (n=7) body				
							17.1% (n=12) antrum				
							50% (n=35) Lesser curvature				
							1.4% (n=1) Greater curvature				

**Table 2:** Comparison of the E-cadherin expression and staining intensity in both groups (case & control)

Staining intensity Groups	Low	High
	**01+**	**2** **+**
Case	20% 28.6% (n=14)(n=20)	51.4% (n=36)
Control	00	100% (n=70)

**Figure 1:**
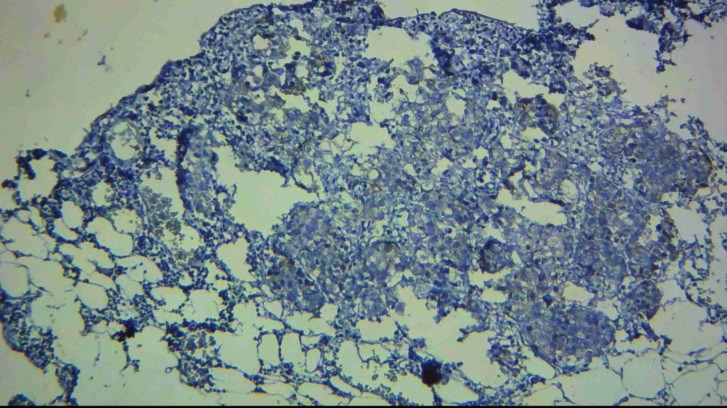
Low staining in the tumoral cells in gastric carcinomas (intestinal type) with E-cadherin marker in IHC staining (magnification 100X)

**Figure 2:**
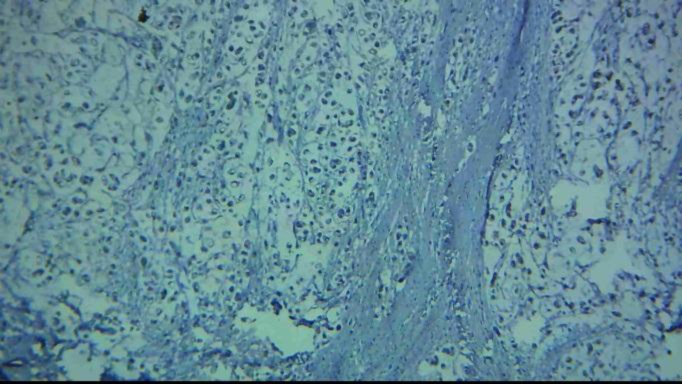
Low staining in the tumoral cells in gastric carcinomas (signet ring type) with E-cadherin marker in IHC staining (magnification 100X)

**Figure 3:**
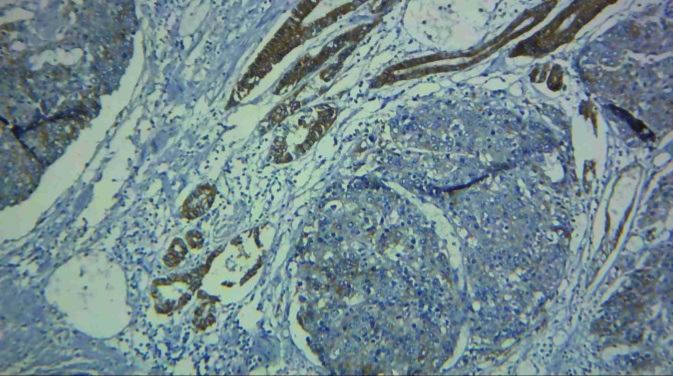
Low staining in the tumoral cells (right) and high staining in tumor- adjacent normal looking tissue (left) with E-Cadherin marker (magnification 100X)

By definition (see Materials and Methods) in the case group, 51.4% (n=36) samples showed high staining (2+) and 48.6% (n=34) revealed low staining (0, 1+), but in the control group all of them showed high staining (Figures 1 -3).

By comparing E-cadherin expression with clinicopathologic features in patients with gastric cancer and statistical analysis of data, a significant correlation between the expression of tumor marker and patients’ age, tumor type, tumor phenotype, depth of invasion, histologic grade, number of involved lymph nodes, and the stage of the disease was observed. (The results have been mentioned in Table 4).

None of the patients were observed to have distant metastases at the time of initial diagnosis. However, to pursue further follow-up, 5 patients showed distant metastases. No significant correlation was seen between distant metastases and the expression of marker.

A significant correlation between histological grade, the number of involved lymph nodes, the stage of disease and the E-cadherin staining intensity in tumor cells was identified. A Significant difference in E-cadher in staining intensity was observed in stage III compared to stages I and II. The number of involved lymph nodes was directly correlated with decreasing in marker staining intensity. The comparison of clinicopathologic parameters of patients with E-cadherin expression has been listed in Tables 3 and 4.

In our study, tumor-adjacent normal tissues were used as control group, and then E-cadherin expression and intensity of staining in these tissues were evaluated. Since the control group was selected from the adjacent normal tissues, both groups were quite matched for sex and age. In order to analyze and compare E-cadherin expression in the two groups, Chi-square test was used and the results showed significant difference between the groups (p-value <0.001).

**Table 3:** The correlation between E- cadherin expression and clinicopathologic parameters in gastric cancer (significant correlation)

Clinicopathologic parameters		E-cadherin expression			**p-value**
		**High**	**low**		
		**>90** **%**	**10-90%** **<10%**		
Age	<55	88.9%(8)	11.1%(1)	0	0.023
	≥ 55	45.9%(28)	31.1%(19)	23%(14)	
Phenotype	intestinal	63.2(%36)	22.8%(13)	14%(8)	<0.001
	diffuse	0	53.8%(7)	46.2%(6)	
Type	adenocarcinoma	54.5%(36)	28.8%(19)	16.7%(11)	0.014
	Signet ring	0	25%(1)	75%(3)	
Depth	T1	50%(1)	50%(1)	0	0.013
	T2	73.7%(14)	21.1%(4)	5.3%(1)	
	T3	42.9%(21)	30.6%(15)	26.5%(13)	
Grade	well	86.4%(19)	13.6%(3)	0	<0.001
	moderate	57.1%(12)	23.8%(5)	19%(4)	
	poor	18.5%(5)	44.4%(12)	37%(10)	
Lymph node	N0	82.1%(23)	14.3%(4)	3.6%(1)	<0.001
	N1	42.1%(8)	31.6%(6)	26.3%(5)	
	N2	21.7%(5)	43.5%(10)	34.8%(8)	
Stage	Ia- Ib	78.6%(11)	21.4%(3)	0	<0.001
	II	78.9%(15)	10.5%(2)	10.5%(2)	
	IIIa- IIIb	27% (10)	40.5%(15)	32.4%(12)	

**Table 4:** The correlation between E-cadherin expression and clinicopathologic parameters in gastric cancer(No significant correlation)

**Clinicopathologic parameters**		**E-cadherin-expression**			**p-value**
		**high**	**low**		
		**>90%**	**10-90%** **<10%**		
Gender	male	53.1%(26)	24.5%(12)	22.4%(11)	0.466
	female	47.6%(10)	38.1%(8)	14.3%(3)	
Location	cardia	41.7%(5)	41.7%(5)	16.7%(2)	0.697
	fundus	33.3%(1)	33.3%(1)	33.3%(1)	
	body	42.9%(3)	28.6%(2)	28.6%(2)	
	antrum	75%(9)	16.7%(2)	8.3%(1)	
	Lesser curvature	51.4%(18)	25.7%(9)	22.9%(8)	
	Grater curvature	0	1.4%(1)	0	
Shape	Ulcerative	53.7%(36)	28.4%(19)	17.9%(12)	0.061
	Infiltrative	0	33.3%(1)	66.7%(2)	
Size	≤ 5	52.3%(23)	29.5%(13)	18.2%(8)	0.658
	5-10	52%(13)	28%(7)	20%(5)	
	>10	0	0	100%(1)	
Neurovascular invasion	yes	44.1%(15)	26.5%(9)	29.4%(10)	0.17
	no	58.3%(21)	30.6%(11)	11.1%(4)	

## Discussion

 E-cadherin is a tumor suppressor gene that is located on chromosome 16, and produces a membrane protein.

E-cadherin is a calcium-mediated membrane molecule that plays an important role in adhesion and differentiation of gastric epithelial cells which is a very important protective mechanism against tumor formation.

Mutation in E-cadherin gene has had different prevalence in various epithelial cancers.^18^^, ^^19^

In this study, 48.6% of cases suffering from gastric cancer showed abnormal expression of E-cadherin in IHC staining. This frequency among cases suffering from gastric cancer was reported 38% in Dr. Anbiaee et al.’s study in Iran,^20^ 49% in Dr. Lazar et al.’s study in Romania,^21^ and 57% in Dr. Chu et al.’s study in China.^17^

These differences may be related to the methods of mutation assessment (expression of E-cadherin by gene methylation or IHC).

In this study, a significant correlation was found between abnormal expression of E-cadherin and tumor grade, stage, depth of invasion and lymph node involvement. These parameters show the invasiveness of tumor. Also, in Dr. Anbiaee et al.’s study in Iran, a correlation between E-cadherin mutation and the grade and lymph node involvement was seen, but this marker was not correlated with depth of invasion.^20^ In Dr. Saad et al.’s study in Egypt, there was a significant correlation between this marker and lymph node involvement and tumor stage, but the depth of invasion and tumor grade were not linked.^22^In Dr. Chu et al.’s study in China, a correlation between depth of invasion and lymph node involvement was seen, but it was not associated with grade.^17^

Although there are different results about the correlation between mutation of E-cadherin, histopathology and tumor invasiveness, most studies indicate that this mutation is associated with more aggressive tumors.

So, E-cadherin mutation can be used as a significant prognostic factor, and the rate of invasive cancers can be decreased by detecting the causes of mutation and preventing them.

In this study, such a significant correlation was seen between the expression of abnormal marker and tumor phenotype and type that was similar to Dr. Lazar et al.’s in Romania.^21^ Also, in Dr. Zhu’s study in China a correlation with tumor type was observed.^13^In Dr. Micu et al.’s study in Romania^23^and Dr. Anbiaee et al.’s study in Iran^20^no significant correlation was found between abnormal expression of marker and tumor type.

Also, in this study a significant correlation was observed between E-cadherin mutation and patients’ age, but this correlation was not seen in other studies.^20^^, ^^21^

In this study, no significant correlation was seen between abnormal expression of marker and sex, tumor location, size, shape and neurovascular invasion. In Dr. Saad et al.’s study in Egypt, a correlation with vascular involvement ^22^ and in Dr. Chu et al.’s study in China a correlation with neuro- vascular invasion was observed.^17^

In this study, no significant correlation was found between abnormal expression of the marker and distant metastasis. In Dr. Saad et al.’s^22^ and also Dr. Chu et al.’s studies,^17^ there was a significant correlation between abnormal E-cadherin expression and metastasis. This difference is probably due to lack of enough time for patient follow-up. 

## CONCLUSION

 This study showed abnormal E-cadherin expression in 48.6% of cases suffering from gastric cancer. Since abnormal expression of E-cadherin is associated with more aggressive gastric tumors, this marker can be used as a negative prognostic factor.

To achieve more conclusive results, we recommend evaluating the correlation between E-cadherin mutation and survival of patients in future studies. Also, we recommend studying the possible environmental factors that may cause mutation in this gene because it is possible to decrease the rate of invasive gastric cancers by detecting these factors and preventing them.
